# Dietary intake of n-3 long-chain polyunsaturated fatty acids and risk of myocardial infarction in coronary artery disease patients with or without diabetes mellitus: a prospective cohort study

**DOI:** 10.1186/1741-7015-11-216

**Published:** 2013-10-08

**Authors:** Elin Strand, Eva R Pedersen, Gard FT Svingen, Hall Schartum-Hansen, Eirik W Rebnord, Bodil Bjørndal, Reinhard Seifert, Pavol Bohov, Klaus Meyer, J Kalervo Hiltunen, Jan E Nordrehaug, Dennis WT Nilsen, Rolf K Berge, Ottar Nygård

**Affiliations:** 1 Department of Clinical Science, University of Bergen, 5021 Bergen, Norway; 2 Department of Heart Disease, Haukeland University Hospital, Bergen, Norway; 3 Bevital AS, Bergen, Norway; 4 Department of Biochemistry and Biocenter Oulu, University of Oulu, Oulu, Finland; 5 MitoHealth Centre for Bioactive Food Components and Prevention of Lifestyle Diseases, Bergen, Norway; 6 Division of Cardiology, Stavanger University Hospital, Stavanger, Norway

**Keywords:** Coronary artery disease, Diabetes, Dietary n-3 fatty acids, Myocardial infarction

## Abstract

**Background:**

A beneficial effect of a high n-3 long-chain polyunsaturated fatty acid (LCPUFA) intake has been observed in heart failure patients, who are frequently insulin resistant. We investigated the potential influence of impaired glucose metabolism on the relation between dietary intake of n-3 LCPUFAs and risk of acute myocardial infarction (AMI) in patients with coronary artery disease.

**Methods:**

This prospective cohort study was based on the Western Norway B-Vitamin Intervention Trial and included 2,378 patients with coronary artery disease with available baseline glycosylated hemoglobin (HbA1c) and dietary data. Patients were sub-grouped as having no diabetes (HbA1c <5.7%), pre-diabetes (HbA1c ≥5.7%), or diabetes (previous diabetes, fasting baseline serum glucose ≥7.0, or non-fasting glucose ≥11.1 mmol/L). AMI risk was evaluated by Cox regression (age and sex adjusted), comparing the upper versus lower tertile of daily dietary n-3 LCPUFA intake.

**Results:**

The participants (80% males) had a mean age of 62 and follow-up of 4.8 years. A high n-3 LCPUFA intake was associated with reduced risk of AMI (hazard ratio 0.38, 95%CI 0.18, 0.80) in diabetes patients (median HbA1c = 7.2%), whereas no association was observed in pre-diabetes patients. In patients without diabetes a high intake tended to be associated with an increased risk (hazard ratio1.45, 95%CI 0.84, 2.53), which was significant for fatal AMI (hazard ratio 4.79, 95%CI 1.05, 21.90) and associated with lower HbA1c (mean ± standard deviation 4.55 ±0.68 versus 4.92 ±0.60, *P* = 0.02). No such differences in HbA1c were observed in those with pre-diabetes or diabetes.

**Conclusions:**

A high intake of n-3 LCPUFAs was associated with a reduced risk of AMI, independent of HbA1c, in diabetic patients, but with an increased risk of fatal AMI and lower HbA1c among patients without impaired glucose metabolism. Further studies should investigate whether patients with diabetes may benefit from having a high intake of n-3 LCPUFAs and whether patients with normal glucose tolerance should be careful with a very high intake of these fatty acids.

**Trial registration:**

This trial is registered at clinicaltrials.gov as NCT00354081.

## Background

Dietary intakes of fish and omega-3 (n-3) long-chain polyunsaturated fatty acids (LCPUFAs), mainly eicosapentaenoic acid (EPA) and docosahexaenoic acid (DHA), have been associated with a reduced risk of cardiovascular disease and mortality [1,2]. A diet rich in these fatty acids (FAs) is recommended in secondary prevention of coronary heart disease [3]. However, two recent meta-analyses of randomized controlled trials, investigating n-3 LCPUFA intake through diet or supplements, failed to demonstrate an overall preventive effect on cardiovascular events [4,5]. Furthermore, a recent large randomized controlled trial among patients at high cardiovascular risk showed no reduction in heart disease or cardiac death after treatment with 1 g/day of n-3 LCPUFA [6].

Randomized trials with n-3 LCPUFA intervention have shown reduced mortality [7] and improved left ventricular systolic function and functional capacity [8,9] among patients with heart failure, who are frequently insulin resistant [10]. In general, patients with diabetes mellitus are at increased risk of cardiovascular disease complications, including acute myocardial infarction (AMI) and mortality, as compared to patients without diabetes [11]. There is, however, conflicting evidence regarding associations between dietary n-3 LCPUFAs and cardiovascular events among patients with diabetes [12-17]. A recently published large randomized controlled trial of 12,536 patients with dysglycemia (ORIGIN), failed to demonstrate a benefit of daily n-3 LCPUFA supplementation [18]. Participants had a baseline dietary n-3 LCPUFA intake of approximately 200 mg/day and median glycosylated hemoglobin (HbA1c) of 6.4%. The intervention group received 1 g/day of n-3 LCPUFAs, while the placebo group received 1 g/day of olive oil. Notably, mortality rate in ORIGIN was higher than could be expected (2.57%) [18] compared to other trials investigating glucose lowering therapy in patients with diabetes. The ACCORD trial (baseline median HbA1c 8.1%) had a mortality rate of 1.41% in the intensive treatment group and demonstrated that aggressive glucose lowering in patients with diabetes, targeting HbA1c <6.0%, may be associated with increased mortality [19]. Based on this, the overall intensive glucose lowering in ORIGIN may have influenced the negative results regarding n-3 LCPUFA supplements.

Our previous investigation on the current population of Norwegian patients with established coronary artery disease (CAD) showed no overall reduction in risk of coronary events with increasing intakes of n-3 LCPUFAs [20]. No sub-group analyses were, however, conducted. Based on the reported beneficial effects in patients with heart failure [7-9], the current investigation evaluated the association of n-3 LCPUFA intake with risk of AMI in patients with no diabetes, pre-diabetes, or established diabetes, respectively, in the patient cohort previously studied. We hypothesized that participants with diabetes mellitus would benefit from a high intake of n-3 LCPUFAs.

## Methods

### Study population

The current investigation was a prospective cohort study based on participants from the Western Norway B-Vitamin Intervention Trial (WENBIT) [21]. In short, this was a clinical trial conducted between 1999 and 2006 at Haukeland University Hospital and Stavanger University Hospital in Western Norway, including 3,090 patients undergoing coronary angiography for suspected CAD, and who were randomized to treatment with B vitamins. Overall, there was no short- or long-term benefits on cardiovascular outcomes or all-cause mortality associated with the study treatment [21].

**Figure 1:**
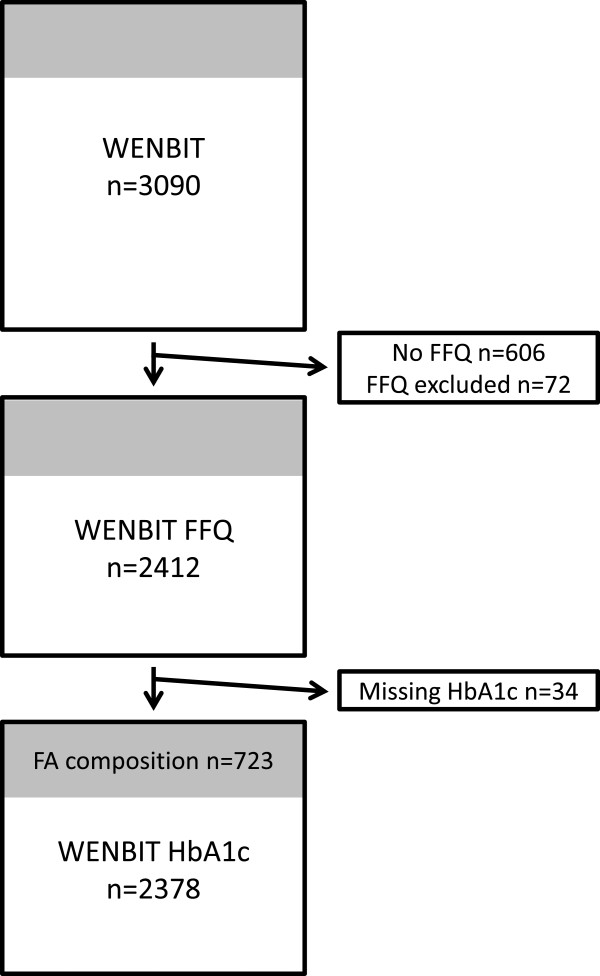
**Flow of randomized patients from WENBIT to WENBIT HbA1c.** WENBIT HbA1c designates the present study population with available dietary and HbA1c data. FFQ, Food Frequency Questionnaire; WENBIT, Western Norway B-Vitamin Intervention Trial.

Figure 1 gives an overview of patient selection for the final inclusion in the present analysis based on 2,484 patients who completed a semi-quantitative food-frequency questionnaire (FFQ) at trial enrolment between 2000 and 2004. Nineteen questionnaires were excluded because they contained more than one blank page. Extreme outliers of dietary intake were excluded based on the assumption that these did not represent their usual daily intakes. These outliers were identified by participants having very low (<3,000 kJ for women and <3,300 kJ for men) or very high (&15,000 kJ for women and &17,500 kJ for men) estimated daily energy intakes (n = 53), leaving 2,412 patients with valid dietary data. Further, 34 individuals were excluded because of missing HbA1c data, leaving 2,378 patients for the final analyses. Serum FA composition was determined in a sub-set of 723 patients.

All participants gave written informed consent. The study protocol was in accordance with the principles of the Declaration of Helsinki, and it was approved by the Regional Committee for Medical Research Ethics, the Norwegian Medicines Agency, and the Data Inspectorate. WENBIT is registered at clinicaltrials.gov as NCT00354081.

### Dietary assessment

An FFQ developed at the Department of Nutrition, University of Oslo, validated against plasma phospholipid n-3 LCPUFA concentrations [22], was given to patients at enrolment and returned by mail to the study center or collected at the first follow-up appointment one month later. A more detailed description of the structure and organization of the 169 food item FFQ and inclusion/exclusion criteria for this sub-study have been given previously [20]. Briefly, the usual daily intake of n-3 LCPUFAs during the last year was estimated based on FFQ reported food items and supplements containing EPA, docosapentaenoic acid (DPA, 22:5n-3), and DHA. Calculations were made by using a database and a software system developed at the Department of Nutrition, University of Oslo (Kostberegningssystem, version 3.2; University of Oslo, Norway) [23].

### Assessment of other covariates

Demographic, clinical, and routine laboratory data were obtained by study personnel at the respective two study centers as previously described [21]. Serum samples were collected before angiography and stored at −80°C until analysis, while standard blood laboratory parameters were analyzed from fresh samples according to routine protocols at their central hospital laboratories. Reagent kits of type Tina-quant® on apolipoprotein A-I (Apo A-I, ver.2), apolipoprotein B (Apo B, ver.2), and C-reactive protein (latex, high sensitive assay) were obtained from Roche Diagnostics (Mannheim, Germany), and serum measurements were done on the Hitachi 917 system (Roche Diagnostics). HbA1c was determined by matrix-assisted laser desorption/ionization time-of-flight mass spectrometry [24] and plasma cotinine by liquid chromatography/tandem mass spectrometry at BEVITAL AS (http://www.bevital.no, Bergen, Norway). Serum FA methyl esters were obtained and analyzed by gas–liquid chromatography as previously described [25]. Smokers included self-reported current smokers, those reported having quit within the last four weeks, and patients with plasma cotinine ≥85 nmol/L. Left ventricular ejection fraction was determined by ventriculography or echocardiography, and values <50% were considered equaling impaired systolic function. Estimated glomerular filtration rate was calculated applying the equation by the Chronic Kidney Disease Epidemiology Collaboration [26]. Extent of CAD was graded as clinically non-significant stenosis (luminal narrowing <50%), or as having single-, double-, or triple vessel disease.

### Endpoints and follow-up

The endpoints in this study were fatal and non-fatal AMI, classified according to the diagnostic criteria of the revised definition of myocardial infarction from 2000 [27]. Procedure-related non-fatal AMI occurring ≤24 hours after coronary angiography, percutaneous coronary intervention (PCI), or coronary artery bypass graft surgery (CABG) were excluded. Information on AMIs was collected from the Western Norway Cardiovascular Registry and from the Norwegian Cause of Death Registry. Endpoints were recorded during in-trial and post-trial follow-up until 31 December 2006, and all events were adjudicated by members of the WENBIT endpoints committee.

### Statistical analyses

Categorization of the participants was based on established diagnostic criteria for no-, pre-, and overt diabetes mellitus [28]. Participants were thus classified as non-diabetic (no prior history of diabetes and HbA1c <5.7%), pre-diabetic (no prior history of diabetes and HbA1c ≥5.7%), and diabetic (previously diagnosed diabetes or fasting baseline serum glucose ≥7.0 or a non-fasting glucose ≥11.1 mmol/L). Means (± standard deviation (SD)) or medians (25^th^, 75^th^ percentile) and proportions of various clinical and biochemical parameters were calculated for selected baseline characteristics and dietary variables within each category. Simple comparisons of continuous variables within or between sub-groups were made by the t-test. Spearman’s rank correlation was used to assess associations between various continuous parameters. The Kolmogorov-Smirnov test was used to examine the continuous FA variables for normal distribution. Variables that were not normally distributed were log-transformed. Estimated marginal means (95% confidence interval (CI)) of FA profile were calculated for each sub-group by one-way analysis of covariance, with adjustments made for age, sex, and statin dose. Post-hoc comparisons were made by using the Tukey honestly significant difference for specifying between-group differences in FA composition as assessed by analysis of covariance.

Participants within each group were ranked into tertiles according to combined daily n-3 LCPUFA (EPA, DPA, and DHA) intake as percentage of total energy (%TE) intake. Survival curves were created for follow-up until the 95^th^ percentile of follow-up time (corresponding to 6.8 years) using the Kaplan-Meier method. Hazard ratios (HRs) and 95% CI were estimated by Cox proportional hazards modeling. Tests for trend were performed using estimated daily n-3 LCPUFA tertiles as a continuous variable in otherwise identical models. The basic model included age and sex. Additional covariates in the multivariate model were selected on the basis of clinical relevance and included the following: fasting (dichotomous), current smoking (dichotomous) [29], extent of CAD (non-significant; single-, double-, or triple-vessel disease), left ventricular ejection fraction (continuous), serum triglycerides (continuous), baseline acute coronary syndrome (dichotomous), baseline PCI (dichotomous), baseline CABG (dichotomous), and randomization to treatment with folic acid or vitamin B6 study medication (dichotomous). Interactions between intake of n-3 LCPUFAs and diabetes were tested by adding interaction product terms in the model. Additional adjustments for the following covariates did not appreciably alter the results and were not included in the final model: body mass index (continuous); current use of statins, β-blockers, angiotensin-converting enzyme inhibitors, angiotensin receptor blockers, metformin, sulfonamides, and insulin (dichotomous for all); history of hypertension (dichotomous); and apolipoprotein A-I and B, HbA1c, or C-reactive protein (continuous for all).

Statistics were performed using IBM SPSS Statistics for Windows, version 19 (SPSS, Chicago, IL, USA) and R version 2.15.2 (R Development Core Team, Vienna, Austria). Two-sided *P*-values <0.05 were considered statistically significant.

## Results

### Baseline characteristics and dietary intakes

At baseline, the mean age of the participants was 61.6 years and 80.4% were men. Further, 84.6% had stable angina pectoris. A total of 46.6% were treated for hypertension and 31.1% were current smokers. A previous myocardial infarction was reported in 41.3%, PCI in 21.5%, and CABG in 14.0% of the patients. On baseline coronary angiography, 11.2% were diagnosed with non-significant CAD, and 32.0% had three-vessel disease. At discharge from hospital, 90.1% of the participants were treated with aspirin, 22.7% with calcium channel blockers, and 9.3% with loop diuretics. A total of 1,577 patients (66.3%) underwent myocardial revascularization with either PCI or CABG. There were 1,012 patients (42.6%) classified as non-diabetic, 1,049 (44.1%) classified as pre-diabetic, and 317 (13.3%) classified as diabetic, of whom 16 (0.7%) had type 1 and 301 (12.7%) type 2 diabetes.

Characteristics of participants in the sub-groups are presented in Table 1. Patients with diabetes (median HbA1c 7.2%) were older (*P* = 0.001), had a higher body mass index (*P* <0.001), and more frequently had hypertension (*P* <0.001). As expected, they had overall higher triglycerides (*P* <0.001) and lower apolipoprotein A-I (*P* <0.001) compared to non- and pre-diabetic participants. Patients with diabetes also had a higher intake of total fat (*P* = 0.02) and monounsaturated fat (*P* = 0.002). There were no differences between the groups regarding intakes of saturated and *trans* fat.

**Table 1:** Baseline characteristics of participants (n = 2,378)

	**Non-diabetes HbA1c <5.7%** **n = 1,012**	**Pre-diabetes HbA1c ≥5.7%** **n = 1,049**	**Diabetes**^**a**^ **n = 317**	***P***^**b**^
Age (y)	61.0 ±9.9^c^	61.8 ±9.5	63.1 ±9.5	0.001
Male sex (n (%))	800 (79.1)	855 (81.5)	256 (80.8)	0.28
Body mass index (kg/m^2^)	26.5 ±3.4	26.7 ±3.6	28.4 ±4.3	<0.001
**Coronary history (n (%))**				
Myocardial infarction	410 (40.5)	432 (41.2)	139 (43.8)	0.34
Percutaneous coronary intervention	209 (20.7)	229 (21.8)	74 (23.3)	0.29
Coronary artery bypass graft surgery	135 (13.3)	150 (14.3)	47 (14.8)	0.44
**Coronary risk factors (n (%))**				
Hypertension^d^	430 (42.5)	461 (43.9)	217 (68.5)	<0.001
Current smoker^e^	313 (30.9)	339 (32.3)	88 (27.8)	0.57
**Serum lipids**				
Triglycerides (mmol/L)	1.73 ±1.28	1.76 ±1.00	2.17 ±1.27	<0.001
Apolipoprotein A-I (g/L)	1.28 ±0.25	1.24 ±0.25	1.22 ±0.26	<0.001
Apolipoprotein B (g/L)	0.90 ±0.25	0.86 ±0.23	0.88 ±0.22	0.004
**Glucose status**				
Glucose (mmol/L)	5.6 ±1.1	5.8 ±1.1	9.8 ±3.4	<0.001
HbA1c (%)	4.9 ±0.6	6.5 ±0.8	7.5 ±1.8	<0.001
**Inflammation markers and renal function**				
C-reactive protein (mg/L)^f^	1.76 (0.85, 3.68)	1.85 (0.89, 4.23)	2.07 (1.00, 4.40)	0.45
Estimated glomerular filtration rate (mL/min)	90.0 ±15.3	90.7 ±14.4	88.8 ±17.5	0.61
**LVEF and severity of coronary artery disease at baseline angiography (n (%))**				
LVEF <50%	89 (8.8)	118 (11.2)	44 (13.9)	0.006
Three-vessel disease	316 (31.2)	330 (31.5)	115 (36.3)	0.19
**Medication at discharge from hospital (n (%))**				
Statins	889 (87.8)	948 (90.4)	280 (88.3)	0.35
β-blockers	800 (79.1)	817 (77.9)	236 (74.4)	0.11
ACE inhibitors/ARBs	263 (26.0)	330 (31.5)	159 (50.2)	<0.001
Metformin	0 (0)	0 (0)	98 (30.9)	0.98
Sulfonamides	0 (0)	0 (0)	79 (24.9)	0.98
Insulin	0 (0)	0 (0)	77 (24.3)	0.98
Other anti-diabetic drugs	0 (0)	0 (0)	4 (1.3)	0.98
**Estimated daily dietary intakes**				
Energy (kJ)	8,790 ±2,560	8,900 ±2,740	8,300 ±2,680	0.06
Total fat (%TE)	31.2 ±5.4	31.4 ±5.4	32.2 ±6.2	0.02
Saturated and *trans* fat (%TE)	11.6 ±2.6	11.6 ±2.6	11.7 ±2.8	0.35
Monounsaturated fat (%TE)	10.1 ±1.9	10.1 ±1.9	10.5 ±2.2	0.002
Polyunsaturated fat (%TE)	7.0 ±1.9	7.0 ±2.0	7.3 ±2.3	0.07
n-6 PUFAs (g)^g^	13.3 ±6.3	13.6 ±6.7	13.1 ±6.6	0.96
n-3 PUFAs (g)^h^	3.23 ±1.54	3.25 ±1.61	3.24 ±1.67	0.89
n-3 LCPUFAs (g)^i^	1.30 ±1.11	1.28 ±1.05	1.32 ±1.06	0.96
n-3 LCPUFAs (%TE)^i^	0.56 ±0.44	0.54 ±0.40	0.60 ±0.46	0.45
**Use of dietary supplements (n (%))**				
Fish oil	167 (16.5)	171 (16.3)	49 (15.5)	0.69
Cod liver oil	272 (26.9)	294 (28.0)	80 (25.2)	0.83
Folic acid	500 (49.4)	523 (49.9)	161 (50.8)	0.68
Vitamin B6	508 (50.2)	545 (52.0)	145 (45.7)	0.44

^a^Diabetes was defined as clinically diagnosed, or as having a fasting glucose ≥7.0 or a non-fasting glucose ≥11.1 mmol/L. ^b^Calculated by using linear regression for continuous variables and logistic regression for binary variables. ^c^Mean ±SD (all such values). ^d^Receiving medical treatment for hypertension. ^e^Current smoker (self-reported, ex-smoker <1 month, or cotinine ≥85 nmol/L). ^f^Values are medians (25th, 75th percentiles). ^g^Composed of linoleic and arachidonic acids. ^h^Composed of α-linolenic acid, EPA, DPA, and DHA. ^i^Composed of EPA, DPA, and DHA. ACE, angiotensin converting enzyme; ARB, angiotensin receptor blocker; DHA, docosahexaenoic acid; DPA, docosapentaenoic acid; EPA, eicosapentaenoic acid; HbA1c, glycosylated hemoglobin; LVEF, left ventricular ejection fraction; n-3 LCPUFAs, n-3 long-chain polyunsaturated fatty acids; PUFAs, polyunsaturated fatty acids; %TE, percentage of total energy.

### Dietary intake of n-3 LCPUFAs and fish

Mean (±SD) daily dietary intakes of n-3 LCPUFAs among all 2,378 participants were 0.43 ±0.24, 1.08 ±0.37, and 2.38 ±1.15 g/day for tertiles 1 to 3 of n-3 LCPUFA, respectively. Adjusted for energy intake, this corresponded to 0.18 ±0.08, 0.45 ±0.09, and 1.03 ±0.40 %TE, respectively. Mean (±SD) intakes of n-3 LCPUFAs (%TE) were 0.56 ±0.44 for non-diabetic, 0.54 ±0.40 for pre-diabetic, and 0.60 ±0.46 for diabetic patients. Intakes were higher among diabetic patients compared to pre-diabetic patients (*P* = 0.04). Tertiles of mean (±SD) daily intakes according to the sub-groups were 0.17 ±0.08, 0.44 ±0.09, and 1.05 ±0.42 %TE for non-diabetic patients; 0.18 ±0.08, 0.44 ±0.08, and 0.98 ±0.38 %TE for pre-diabetic patients; and 0.19 ±0.08, 0.48 ±0.11, and 1.12 ±0.42 %TE for patients with diabetes. Total daily fish intake (mean ±SD) in tertiles 1 to 3 was 47.7 ±19.0, 98.0 ±13.7, and 180.7 ±62.1 g/day, respectively.

### Serum fatty acid profile

FA profile in serum from a sub-cohort of 723 patients was used to determine whether estimated dietary intake of FAs was reflected in serum. We observed a strong association between reported intake and serum total n-3 LCPUFAs (Spearman’s rho = 0.515, *P* <0.001). Table 2 shows main serum FA profile in percentage by weight (wt%) of total FAs in sub-groups of patients with no diabetes (n=380), pre-diabetes (n = 259), and diabetes (n = 84). When adjusted for age, sex, and statin dose, serum total FAs (mg/L) were borderline significantly higher in the diabetic group as compared to those with pre-diabetes (Tukey honestly significant difference, *P* = 0.05). Furthermore, serum saturated FAs were higher (wt%) while n-6 PUFAs were lower in patients with diabetes compared to those with no diabetes and pre-diabetes (Tukey honestly significant difference, *P* <0.05 for all between-group comparisons). There was no difference in total or individual n-3 LCPUFAs between the three sub-groups.

**Table 2:** Serum fatty acid profile in percentage by weight (wt%) measured in 723 participants

	**Non-diabetes HbA1c <5.7%** **n = 380**	**Pre-diabetes HbA1c ≥5.7%** **n = 259**	**Diabetes**^**a**^ **n = 84**	***P***^**b**^
Total FAs (mg/L)	3,790 (3,680 to 3,900)^c^	3,680 (3,560 to 3,810)	4,050 (3,820 to 4,300)	0.02
Saturated FAs	33.2 (32.9 to 33.4)	33.2 (32.9 to 33.5)	34.0 (33.5 to 34.5)	0.01
Monounsaturated FAs	22.9 (22.5 to 23.2)	23.2 (22.7 to 23.6)	24.0 (23.2 to 24.8)	0.03
n-6 PUFAs	35.0 (34.4 to 35.5)	34.5 (33.9 to 35.2)	32.8 (31.8 to 33.9)	0.002
n-3 PUFAs	7.2 (7.0 to 7.5)	7.4 (7.1 to 7.8)	7.2 (6.6 to 7.8)	0.61
α-Linolenic acid	0.71 (0.69 to 0.73)	0.73 (0.70 to 0.75)	0.72 (0.68 to 0.77)	0.38
Eicosapentaenoic acid	1.80 (1.68 to 1.92)	1.91 (1.76 to 2.07)	1.78 (1.54 to 2.05)	0.49
Docosapentaenoic acid (n-3)	0.62 (0.60 to 0.63)	0.63 (0.61 to 0.65)	0.62 (0.58 to 0.65)	0.55
Docosahexaenoic acid	3.69 (3.56 to 3.82)	3.73 (3.58 to 3.90)	3.63 (3.37 to 3.91)	0.79
n-3 PUFA / n-6 PUFA ratio	0.21 (0.20 to 0.22)	0.22 (0.20 to 0.23)	0.22 (0.20 to 0.24)	0.41

^a^Diabetes was defined as clinically diagnosed, or as having a fasting glucose ≥7.0 or a non-fasting glucose ≥11.1 mmol/L. ^b^Calculated by using one-way analysis of covariance, after log transformation, adjusted for age, sex, and statin dose. ^c^Estimated marginal means; 95% CI in parentheses (all such values). FAs, fatty acids; HbA1c, glycosylated hemoglobin; PUFAs, polyunsaturated fatty acids.

### Follow-up and events

Mean (±SD) length of follow-up was 4.8 ±1.4 years. A total of 208 participants (8.7%) experienced a fatal or non-fatal AMI. The incidence of AMI was 7.6% for non-diabetic, 8.4% for pre-diabetic, and 13.6% for diabetic patients.

### Intake of n-3 LCPUFAs and risk of acute myocardial infarction

**Figure 2:**
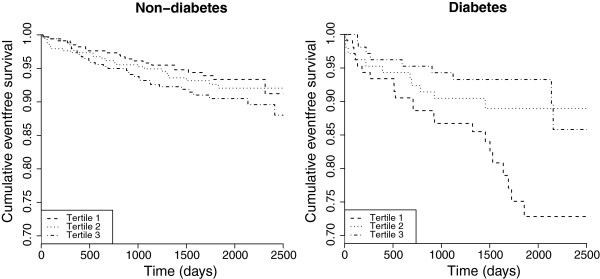
**Kaplan-Meier plot illustrating survival in patients with or without diabetes mellitus.** Survival plot showing time to an acute myocardial infarction in each tertile of n-3 LCPUFA intakes (designated as Tertile 1, 2, and 3) in patients without diabetes (HbA1c <5.7%) (left panel) and with diabetes (right panel). Estimates were based on follow-up until the 95^th^ percentile (6.8 years).

Figure 2 illustrates the crude event-free survival time from AMI among patients with or without diabetes in tertiles of n-3 LCPUFA intake. The age and sex- and multivariate-adjusted HRs for AMI according to tertiles of n-3 LCPUFA consumption (%TE) in sub-groups, as estimated by Cox proportional hazards modeling, are presented in Table 3. Compared to the lower tertile, the multivariate adjusted risk of experiencing an AMI was non-significantly increased by 55% among patients without diabetes, having intakes corresponding to the upper tertile (*P* = 0.13). There was no association between n-3 LCPUFA consumption and risk of AMI in patients with pre-diabetes, and restricting the analysis to patients with HbA1c ≥6.5% (n = 423) provided similar results (data not shown). Among patients with diabetes, there was a 62% risk reduction of experiencing an AMI in the upper compared to the lower tertile of n-3 LCPUFA intakes in multivariate analysis (*P* = 0.02), and there was also a dose–response effect (*P* for trend = 0.01). Adding all patients with HbA1c ≥6.5% to the diabetes group clearly attenuated the associations, providing a multivariate adjusted HR (95% CI) of 0.71 (0.40, 1.26) in the upper versus lower tertile of n-3 LCPUFA intakes and no dose–response relationship. Estimates based on mg/day amounts of n-3 LCPUFAs were very similar and did not introduce any material changes to the percentage of total energy findings (data not shown). There were no clear associations between total fish intake and AMI risk (Table 3).

**Table 3:** Risk of total acute myocardial infarction by dietary n-3 LCPUFA (as a percentage of total energy intake) and fish (grams) tertiles

	**Non-diabetes (HbA1c <5.7%, n = 1,012)**		**Pre-diabetes (HbA1c ≥5.7%, n = 1,049)**		**Diabetes (n = 317)**^**a**^	
	**Age/sex adjusted**	**Multivariate**^**b**^	**Age/sex adjusted**	**Multivariate**^**b**^	**Age/sex adjusted**	**Multivariate**^**b**^
Number of events	77		88		43	
**n-3 LCPUFA**						
Tertile 1^c^	1.00	1.00	1.00	1.00	1.00	1.00
Tertile 2^d^	1.12 (0.63, 2.02)	0.93 (0.49, 1.77)	1.16 (0.69 to 1.95)	1.20 (0.70, 2.06)	0.43 (0.21, 0.88)	0.48 (0.22, 1.06)
Tertile 3^e^	1.45 (0.84, 2.53)	1.55 (0.87, 2.76)	1.16 (0.69, 1.95)	1.33 (0.77, 2.28)	0.38 (0.18, 0.80)	0.38 (0.17, 0.83)
*P* for trend	0.17	0.11	0.59	0.30	0.007	0.01
**Total fish**						
Tertile 1^f^	1.00	1.00	1.00	1.00	1.00	1.00
Tertile 2^g^	1.68 (0.97, 2.89)	1.51 (0.84, 2.72)	0.85 (0.50, 1.44)	0.88 (0.51, 1.52)	0.87 (0.44, 1.71)	0.94 (0.45, 1.98)
Tertile 3^h^	1.02 (0.55, 1.88)	1.19 (0.63, 2.27)	1.05 (0.63, 1.74)	1.10 (0.65, 1.85)	0.56 (0.26, 1.24)	0.66 (0.29, 1.53)
*P* for trend	0.96	0.60	0.84	0.70	0.16	0.34

Hazard ratios and 95% confidence intervals were calculated using Cox proportional hazards. ^a^Diabetes was defined as clinically diagnosed, or as having a fasting glucose ≥7.0 or a non-fasting glucose ≥11.1 mmol/L. ^b^Multivariate model adjusted for age (continuous), sex, fasting (yes or no), current smoker (yes or no), extent of coronary artery disease (non-significant; single, double, or triple vessel), left ventricular ejection fraction (continuous), serum triglycerides (continuous), baseline acute coronary syndrome (yes or no), baseline percutaneous coronary intervention (yes or no), baseline coronary artery bypass graft surgery (yes or no), and treatment with folic acid or vitamin B6 supplements (yes or no). ^c^n = 337 in non-diabetic, n = 349 in pre-diabetic, and n = 105 in diabetic patients. ^d^n = 338 in non-diabetic, n = 350 in pre-diabetic, and n = 106 in diabetic patients. ^e^n = 337 in non-diabetic, n = 350 in pre-diabetic, and n = 106 in diabetic patients. ^f^n = 339 in non-diabetic, n = 354 in pre-diabetic, and n = 105 in diabetic patients. ^g^n = 335 in non-diabetic, n = 342 in pre-diabetic, and n = 108 in diabetic patients. ^h^n = 338 in non-diabetic, n = 353 in pre-diabetic, and n = 104 in diabetic patients. HbA1c, glycosylated hemoglobin; n-3 LCPUFA, n-3 long-chain polyunsaturated fatty acids (eicosapentaenoic acid, docosapentaenoic acid, and docosahexaenoic acid).

Conventional criteria on diagnosing diabetes by blood glucose rely on two separate measurements [28]. Since 59 patients were included in the diabetes group based on baseline glucose from one single measurement, we did a separate sensitivity analysis among the 258 patients who were diagnosed with diabetes prior to baseline examination, and this did not change the results (data not shown). Because patients with diabetes tended to have an increased n-3 LCPUFA intake compared to the others, we repeated the analysis on the diabetes sub-group using tertiles based on the total population. This analysis provided similar results (data not shown). There was an interaction between diabetes and n-3 LCPUFA in a multivariate adjusted interaction analysis (*P* for interaction = 0.02). Adjusting for study treatment with B vitamins according to the WENBIT study design did not materially affect any of the results.

We also performed stratified analyses according to fatal- and non-fatal AMI as outcomes (Table 4). Among patients without diabetes, HR (95% CI) for fatal AMI was 4.79 (1.05, 21.90) in the upper versus lower tertile of n-3 LCPUFA intake (*P* for trend = 0.02). In patients with diabetes, HR (95% CI) for fatal AMI was 0.22 (0.06, 0.81) in tertile 3 compared to tertile 1 (*P* for trend = 0.02). For non-fatal AMI, there was no change in risk among patients without diabetes, but a non-significant trend towards a reduced risk with increasing intakes among patients with diabetes. Due to the low number of events in each sub-group, the multivariate model did not converge for separate fatal- and non-fatal AMIs. Thus, only age- and sex adjusted HRs were presented for these separate endpoints (Table 4).

**Table 4:** Risk of acute myocardial infarction (separate fatal and non-fatal) by dietary n-3 LCPUFA tertiles (as a percentage of total energy intake)

	**Non-diabetes (HbA1c <5.7%, n = 1,012)**	**Pre-diabetes (HbA1c ≥5.7%, n = 1,049)**	**Diabetes (n = 317)**^**a**^
**Fatal AMI (events)**	**16**	**20**	**18**
Tertile 1^b^	1.00	1.00	1.00
Tertile 2^c^	1.98 (0.36, 10.82)	1.85 (0.56, 6.15)	0.39 (0.13, 1.15)
Tertile 3^d^	4.79 (1.05, 21.90)	1.84 (0.56, 6.13)	0.22 (0.06, 0.81)
*P* for trend	0.02	0.34	0.02
**Non-fatal AMI (events)**	**61**	**68**	**25**
Tertile 1^b^	1.00	1.00	1.00
Tertile 2^c^	1.03 (0.55 to 1.93)	1.04 (0.58 to 1.87)	0.45 (0.17 to 1.20)
Tertile 3^d^	1.10 (0.60 to 2.04)	1.04 (0.58 to 1.86)	0.52 (0.20 to 1.32)
*P* for trend	0.75	0.91	0.14

Hazard ratios and 95% confidence intervals were calculated using Cox proportional hazards. The model was adjusted for age (continuous) and sex. ^a^Diabetes was defined as clinically diagnosed, or as having a fasting glucose ≥7.0 or a non-fasting glucose ≥11.1 mmol/L. ^b^n = 337 in non-diabetic, n = 349 in pre-diabetic, and n = 105 in diabetic patients. ^c^n = 338 in non-diabetic, n = 350 in pre-diabetic, and n = 106 in diabetic patients. ^d^n = 337 in non-diabetic, n = 350 in pre-diabetic, and n = 106 in diabetic patients. HbA1c, glycosylated hemoglobin; n-3 LCPUFA, n-3 long-chain polyunsaturated fatty acids (eicosapentaenoic acid, docosapentaenoic acid, and docosahexaenoic acid).

*Post hoc* comparisons within the non-diabetic group revealed that HbA1c was lower in tertiles 2 (*P* = 0.008) and 3 (*P* = 0.01), compared to tertile 1 of n-3 LCPUFA intakes (mean ±SD 4.87 ±0.62 and 4.87 ±0.65 versus 4.99 ±0.54). Furthermore, patients without diabetes who experienced an AMI did also have a lower HbA1c than those who did not have an AMI event (mean ±SD 4.77 ±0.63 versus 4.92 ±0.60, *P* = 0.04). This association was more pronounced among those who had a fatal AMI event (mean ±SD 4.55 ±0.68 versus 4.92 ±0.60, *P* = 0.02). No such differences were observed in those with pre-diabetes or diabetes.

## Discussion

The main finding in this observational cohort study among patients with established CAD was that a high intake of n-3 LCPUFAs was associated with a reduced risk of total AMI, independent of HbA1c, in patients with diabetes, but with an increased risk of fatal AMI and with lower HbA1c in those without impaired glucose metabolism.

One of the strengths of this study was its large, well-characterized population with long-term follow-up and extensive dietary information. Data are scarce on the association between dietary intake of n-3 LCPUFA and AMI in statin-treated patients with CAD, since most previous studies have based their results on circulating n-3 LCPUFAs and many investigations have been performed in groups that did not receive statins. This study helps address this gap in data. All endpoints in the current study were validated by a committee blinded to the dietary information.

This study also had certain limitations. Our study had limited power to detect significant effects due to the smaller sample size of sub-groups and the low event rate primarily in the non-diabetes group. Furthermore, FFQs were not checked for errors when received at the study center, and participants with extreme values or partially missing reported intakes were excluded ahead of this sub-study. The remaining data were considered valid, as dietary intakes were comparable to previous surveys in the region using the same questionnaire [30]. Information on dietary habits was collected at baseline, reflecting the average intake during the past year, whereas no information on dietary habits was collected during follow-up. Thus, there is a possibility of a regression dilution bias, which probably would have strengthened rather than attenuated the results. Despite careful adjustments for available important covariates, prospective cohort studies are typically limited by confounding from both unmeasured and inappropriately measured factors.

Similar to our previous investigation [20], a study among post-AMI patients did not report any overall relations between n-3 LCPUFAs and cardiovascular events [31]. However, in accordance with our current results, a *post hoc* analysis among participants with diabetes revealed a strong decline in ventricular arrhythmia-related events and fatal AMI with increased n-3 LCPUFA intake [32]. Other studies have also indicated that a high intake may reduce the risk of non-fatal AMI [33-36]. A recent study concluded that n-3 PUFA supplementation might have the ability to reduce cardiovascular events in patients not treated with statins [37], whereas no additional benefits were observed among statin users [37]. The majority of patients in the present study were treated with statins. Thus, our data indicate that n-3 LCPUFA intake is associated with AMI risk, and particularly fatal events, also in statin users.

The main question is whether our findings are observed by chance or report real effects of n-3 LCPUFA intake. No effects of n-3 LCPUFA supplementation were revealed in the large ORIGIN study among dysglycemic patients [18]. These participants had a low baseline dietary intake of n-3 LCPUFAs, and even after receiving study treatment, daily n-3 LCPUFA intake was only about half compared to those having the highest intakes in our population. Additionally, olive oil was used as placebo in ORIGIN as well as in several other randomized trials [38]. This oil contains various FAs, and its use as placebo may thus have influenced the results. Furthermore, participants in ORIGIN had a median HbA1c of 6.4%, whereas patients with diabetes in the current study had a median HbA1c of 7.2%. Patients with HbA1c ≥9.0% were excluded from ORIGIN, whereas those with fasting plasma glucose ≥6.1 mmol/L were included. In a separate analysis in our cohort, we included all patients with HbA1c ≥6.5% together with the patients diagnosed with diabetes, which attenuated the results. Based on these considerations, the diabetes group in our study differs clearly from the dysglycemic patients in ORIGIN, having a similar average HbA1c as in our sub-group of patients with pre-diabetes where no association with n-3 LCPUFA was observed. Notably, the ACCORD study among patients with diabetes having baseline median HbA1c 8.1% demonstrated adverse effects with an increased mortality after aggressive glucose lowering treatment [19]. Thus, the overall intensive glucose lowering in ORIGIN may also have influenced outcome following n-3 LCPUFA supplementation.

Dietary n-3 LCPUFAs are predominantly present in fish and seafood, in which oily fish is a major source. Existing international guidelines recommend an n-3 LCPUFA intake of at least 250 mg/day or two servings of oily fish per week [39]. Despite the observed associations between dietary n-3 LCPUFAs and AMI risk, no clear associations could be seen for fish intake. This might be explained by a frequent use of cod liver and/or fish oils among those having the highest intakes of n-3 LCPUFAs, with 70% using such supplements in the upper tertile (data not shown). Even though n-3 LCPUFA supplements like cod liver and fish oils were frequently used, fish consumption was also relatively high in this cohort. Thus, we cannot exclude that other fish ingredients may have influenced the results.

Another prominent finding in the present study was the strong association between a high intake of n-3 LCPUFAs and fatal AMI in patients without diabetes with HbA1c <5.7%. Dietary intake of high-dose n-3 LCPUFA has also in some previous studies been associated with adverse effects. A study in South Wales on male patients with angina pectoris revealed an increased risk of cardiac death among participants advised to eat oily fish or fish oil capsules [40]. Furthermore, a canine model showed a pro-arrhythmic effect after high-dose n-3 PUFA supplementation in dogs not originally vulnerable to ischemia [41].

Through a *post hoc* comparison within the non-diabetic group, we demonstrated an overall lower HbA1c in those having the highest intake of n-3 LCPUFAs and in those who experienced a fatal AMI compared to the other individuals within the non-diabetic group. A previous prospective cohort study demonstrated an increased risk of all-cause death among individuals with HbA1c <5.0% (no diabetes) [42]. A link has been demonstrated between hypoglycemia, endothelial dysfunction, and increased oxidative stress [43], which could produce a certain metabolic profile. Accordingly, high-dose fish oil supplementation has also been associated with increased oxidative damage in rats [44]. In view of our own observations, it is curious to note that when fasted, dienoyl-CoA reductase (*Decr*) null mutant mice develop hypoglycemia and accumulate PUFAs in their tissues, indicating an impaired mitochondrial β-oxidation [45]. This may further suggest a reverse relationship between PUFAs and blood glucose in fasted individuals. Our findings could imply that high-dose n-3 LCPUFA consumption among patients without diabetes or impairment of glucose tolerance might reduce overall blood glucose and increase the risk of fatal AMI. Notably, no association was seen between HbA1c and the n-3 LCPUFA effects among patients with diabetes.

Altogether, additional studies are needed to validate our findings and further elucidate mechanisms behind the observed associations. Based on future research, it should be concluded whether it is time for a reassessment of the current dietary advices on n-3 LCPUFAs in secondary prevention of CAD [3].

## Conclusions

In this cohort of patients with established CAD, a high intake of n-3 LCPUFAs was associated with reduced risk of AMI, independent of HbA1c, in patients with diabetes. In patients without diabetes, a high intake was associated with increased risk of fatal AMI and lower HbA1c. These findings should motivate further studies on potential beneficial or adverse effects of a high n-3 LCPUFA intake in sub-groups of patients with CAD.

## Abbreviations

%TE: Percentage of total energy; AMI: Acute myocardial infarction; CABG: Coronary artery bypass graft surgery; CAD: Coronary artery disease; CI: Confidence interval; DHA: Docosahexaenoic acid; DPA: Docosapentaenoic acid (n-3); EPA: Eicosapentaenoic acid; FA: Fatty acid; FFQ: Food frequency questionnaire; HbA1c: Glycosylated hemoglobin; HR: Hazard ratio; LCPUFA: Long-chain polyunsaturated fatty acid; n-3: Omega-3; PCI: Percutaneous coronary intervention; SD: Standard deviation; WENBIT: Western Norway B-Vitamin Intervention Trial; wt%: Percentage by weight.

## Competing interests

The authors declare that they have no competing interests.

## Authors’ contributions

RKB and ON designed research; ES, ERP, GFTS, HSH, EWR, PB, KM, and ON conducted research; ES, RS, PB, KM analyzed data or performed statistical analysis; ES and ON wrote the paper; ES had primary responsibility for final content; ES, BB, JKH, RKB, and ON interpreted data; ES, GFTS, HSH, EWR, BB, RS, PB, JKH, JEN, DWTN, RKB, and ON critically revised the manuscript. All listed authors take responsibility for all aspects of the reliability and freedom from bias of the data presented and their discussed interpretation. All authors read and approved the final manuscript.

## Pre-publication history

The pre-publication history for this paper can be accessed here:

http://www.biomedcentral.com/1741-7015/11/216/prepub
